# Extract from a Lecture on the Physiological and Remedial Effects of Increased Pressure of the Atmosphere

**Published:** 1867-01

**Authors:** Charles A. Lee

**Affiliations:** Professor of Hygiene, etc.


BUFFALO

### Medical and Surgical Journal.

VOL. VI. JANUARY, 1867. No. 6.

### Original Communications.

ART. I—*Extract from a Lecture on the Physiological and Remedial Effects of Increased Pressure of the Atmosphere.* By Charles A. Lee, M. D., *Professor of Hygiene, etc.*

Having considered somewhat in detail some of the meteorological conditions which affect the inhabitants of our globe, let us consider, briefly, the physiological and curative effects of increased pressure of atmospheric air upon the human body. Assuming that the surface of the body of an adult, of medium size, is about fifteen square feet, or 2160 inches, when the barometer stands at thirty-one inches, the pressure upon each square inch of surface will exceed fifteen pounds, and on the whole surface, 33,684 pounds; but when the barometer stands at twenty-eight inches, this will be reduced to about fourteen pounds on each square inch, and to thirty, 622 pounds on the entire surface, making a difference of about 3062 pounds. This pressure is not perceptible, inasmuch as action and re-action are equal; the pressure of the air in any one direction must be exactly counterbalanced by an equal pressure in the opposite direction, and all injurious effects are prevented by the elasticity of the solids, liquids and gases, that enter into the composition of the body. The influence of increased pressure of the atmosphere upon the body, was first noticed in experiments made with the diving-bell; which, as formerly constructed, condensed the air in proportion to the depth of water to which it descended; thus subjecting the persons within to the pressure, perhaps, of several atmospheres. The physiological effects of such pressure were carefully noted, from time to time, especially the pain in the ears, which was often very severe; and it was also noticed that the respiration and pulse were both reduced in frequency. It was also observed that persons laboring under a considerable degree of deafness, were able to hear, while in the diving-bell, much better than in the open air.

Particular attention, however, was not directed to the subject till the year 1841, when an article was published at Berlin, in the "*Archives for Mineralogy, Geology and Mining*," giving a very interesting account of the effects of highly condensed air upon the human body, as observed in an apparatus used in mining. It appears, that, in order to keep out the influx of water from a mining-shaft, a cylinder of sheet-iron seventy feet long and three and one-half feet in diameter, was passed down through the land, till it reached the bed of coal. At the upper end of the cylinder was a box, with two valves large enough for a man to pass in and out, without allowing air to escape, and the air was forced into the cylinder by air pumps, worked by a steam engine. In this case similar phenomena were observed, as had already been noticed in those who had descended into water in the diving-bell. On first entering, before the balance between the internal and external pressure was restored, there was severe pain in the ears, which gradually ceased, as soon as an equilibrium was established. There was, however, a great difference in this respect in different persons. In some individuals the pain was very slight, in others very severe. Some only experienced it when they came out into a thinner, or less dense atmosphere. The effect was not uniform, even upon the same persons, being modified very essentially by the state of the general health at the time, and other conditions not easily ascertained. Although able to talk and sing with greater ease than ordinary, under a pressure of three atmospheres, yet when the degree of condensation reached that point, the workmen were unable to whistle. This has been explained in the following manner: In whistling, we force a stream of air through a small aperture of the lips; the fluid thus passing this small aperture, becomes condensed, and produces the sound; but under a pressure of three atmospheres, the air is already so condensed that the ordinary effort which the muscles of the cheeks and lips, and the respiratory organs make in whistling, is not sufficient to compress the air any further, and consequently there is an inability of producing any sound. Another phenomena observed was, that all sounds appeared to be nasal.

It does not appear that any decided effects were produced upon respiration by exposure of the lungs to this greatly increased pressure, except a slightly reduced number of respirations in a minute; although it is stated that in ascending the shaft, yet filled with condensed air, the workmen did not lose their breath, nor become short of breath to the same degree as when making a similar ascent in common atmospheric air. This may, perhaps, be satisfactorily explained, from the fact, that as the same volume of condensed air contains a greater amount of oxygen than the same volume of uncondensed air, the lungs will consequently require, in proportion to the condensation, a smaller volume of air to effect the due oxygenation and decarbonization of the blood, and, of course, do not require to be expanded as much as when breathing uncompressed air; consequently the action of the respiratory muscles need not be as energetic. How much may be owing to this, and how much to the direct effects of pressure on the circulation cannot be definitely ascertained. No such violent effects, however, are stated to have occurred, as have been witnessed in some experiments made with the diving-bell, where, in descending to great depths, (causing a pressure, probably, much exceeding that of three atmospheres, the highest degree mentioned as occurring in the cylinder,) blood flowed from the lungs, nostrils and ears, and the tympanum of the ear was ruptured.

Another circumstance, worthy of note in this case, as well as that of the diving-bell, is, that the workmen gradually became accustomed to the pressure, so that after awhile they suffered comparatively but little inconvenience. In accordance with this fact M. Triger ("*Annales de Hygiene*," 1845) states that in some mining operations, in which he was concerned, he subjected individuals to a pressure of about three atmospheres, and although they suffered pain and inconvenience at first, they soon became accustomed to this degree of condensation. The same fact was observed also, as has been often witnessed in the diving-bell, namely: that deaf persons could hear very distinctly in the condensed air, and in some instances could hear much better than some who were not deaf.

If we compare these effects with those we have described as produced by greatly diminished pressure, as in ascending very lofty mountains, or to a height of many thousand feet in balloons, namely: vertigo and dizziness, hæmorrhages from the nose and mouth, breathlessness, hurried pulse, and quickened respiration, etc., we shall find that the dangers and inconveniences experienced from compressed air, to the extent of three atmospheres, are far less in degree than diminished pressure to the same amount. It is, however, to be recollected that in ascending very lofty mountains, much of the danger and inconvenience is to be attributed to the conjoined influence of muscular action and a rarified atmosphere, and not to the latter alone; inasmuch as balloonists, in ascending to even much greater heights, do not often suffer in an equal degree; and, sometimes, as in the case of Messrs. Green and Rush, who, in 1838, went up to a height of 29,000 feet, or five miles, the suffering, except from cold, was comparatively slight. It is very possible, however, that the benumbing effects of cold, which was very considerable, may have prevented the consciousness of other kinds of suffering. Still the Abbe Ferra, who lived in Italy, states that none but invalids are incommoded in ascending Mt. Ætna. Here, however, the height is inconsiderable, compared with that of the Himmalayas; in the ascent of which, all travelers who have reached the top, describe the physical effects as very strongly marked. The general statement will doubtless hold true, that the influence of the atmosphere in producing morbid sensations or conditions of body, through its changes of weight, unless they are very great, is limited in extent, and usually slight, where the individual exposed to them is in a good state of health; the changes being usually gradual, giving time for the organs to adapt themselves to the range of barometrical variation; and that it is only when they are sudden and extreme, that any injurious consequences are apt to follow. A sudden alteration of balance between the external and internal parts of the body would necessarily affect the vascular system, and derange the circulation, which, to a certain extent, is regulated by mechanical principles. Common observations with the air-pump and cupping-glasses show how readily these vascular textures and the contained fluids yield to such a change of balance, and it is not therefore strange that we should find hæmorrhages of various kinds, especially in persons predisposed to them, from a sudden fluctuation in the weight of the atmosphere. Indeed meteorological statistics compared with accurate meteorological observations lend great support to the belief that hæmorrhages, apoplexy and paralysis are more liable to occur during a low state of the barometer than at the opposite degree. It has been noticed that highly sensitive and nervous persons experience a change in their sensations and feelings upon a sudden alteration in the weight of the atmosphere, which, perhaps, may be a compound effect of the changes in the circulation, in which the sensorium, the lungs, and the muscular system all participate.

But to return to the subject of *compressed air* and its effects on the body. Soon after the publication in the "*French Annals of Hygiene*," (1844) of the effects above described as observed in mining operations, the attention of the medical profession was drawn more particularly to it as a remedial agent in different diseases. Among others, M. Pravas, a distinguished French physician, made many experiments and observations in regard to the effects of atmospheric condensation, and published two elaborate Essays* as early as 1841, upon the subject, which have been translated into English, and published in London some two or three years ago. In the first of these Essays he describes the good effects from its use, associated with gymnastics, in the treatment of rickets, strumous and spasmodic affections and catarrhal deafness. He speaks, also, of its salutary operation in weakness of the lungs, by its nursing their vitality, increasing the richness of the blood, etc. This writer, as well as M. Merat,† has directed attention to another mode of applying compressed air, namely: by inhalation; which, it is stated, has proved very beneficial in diseases of the organs of voice from relaxation of tissue and want of tone in the parts, attended with more or less congestion, and

* Memoire sur l'emplai du bain d' air comprimé se.

† "Dict, univer. de Mat. Med. et de Therap. Gen." (Supplement.)

accumulation of mucus; the period of inspiration recommended being from one to three hours daily.

In consequence, probably, of the publication of the works above mentioned, several establishments were afterwards opened for the treatment of disease by this method, especially in Germany, as at Berlin, Leipsic and Stuttgard, and during the last ten years at numerous other places in that country. During the same period the German medical periodicals have been much occupied with this subject; particularly "Schmidt's Jahr Bücher," in which many able articles have appeared relating to it, setting forth its advantages as well as disadvantages, and the dangers which may attend it. In the same journal for 1863, is an advertisement by a mechanic, offering to furnish a complete apparatus for compressing air, worked by a hand-pump, for the price of two hundred dollars and upwards. Many of these, I am informed, have been sold and distributed throughout the kingdom, and are probably now in use. The treatment by this method in Europe has always been in the hands of the regular faculty, and recognized as a legitimate and scientific mode of practice. In 1862, when in Lyons, in France, I found an extensive establishment of this kind, which enjoyed a wide-spread reputation. In Paris it is believed there are several.

The history of the treatment of disease by compressed air in this country, so far as I am at present informed, is the following: Some six or seven years ago an uneducated layman got up an establishment for this purpose at Ashawa, C. W., where it was carried on for four or five years, with varying success; when about two years since it was removed to Toronto. Here it came under the charge of a Mr. Ware, also a layman, and many persons were attracted thither for treatment for a year or two; several from this city; some of whom represent to me that they were considerably benefited, and some that they were entirely cured. A few months since, from some cause the establishment was broken up, and another soon after was opened by a company as a joint-stock operation, in Rochester, N. Y. While in Toronto, as I am incredibly informed, it was patronized by some of the medical fraternity of the college at that place, and other regular members of the profession, who sent not only patients thither for treatment, but also in some cases members of their own families. Since the establishment has been in operation at Rochester quite a large number of persons have undergone the treatment; some from the large cities of the union, and several from Buffalo, there having been through the last summer some fifty or sixty, who were trying the treatment at the same time. So far, I believe, it has been entirely in charge of practitioners of the homœophathic school; and at no time, or in any place in the United States has the treatment by compressed air, so far as I know, been in charge of regularly educated members of the medical profession.

A wealthy citizen of Buffalo, subject to severe attacks of spasmodic asthma, having experienced decided temporary relief at the establishment at Rochester, is now erecting a more extensive and complete one in this city, probably the most perfect as well as extensive ever established in any country, partly for the benefit of the treatment in his own case, and partly as a pecuniary investment. This is to contain, in addition to the compressed air bath, the usual conveniences for hydropathic treatment; with sulphur baths, from a native sulphur spring in the vicinity; the Turkish hot-air bath, etc. It is to be hoped it may be placed under the management of an able, scientific practitioner.

This *compressed air-bath* will, when completed, consist essentially of an air-tight room, or rooms, made hermetrically tight by a continuous lining of sheet-iron; each room being sufficiently large as to accommodate eight or ten persons at the same time, with a door for entrance, composed mainly of thick plate glass, and the pressure so controlled by valves as to be easily regulated to any degree of density required. The air is to be forced by a steam engine through a reservoir of pure water, which is being constantly renewed, so that all impurities contained in the atmosphere will be effectually got rid of; and thus it will enter the air-chamber thro' a tube connected therewith, perfectly pure; whence it is to pass off through a regulating or safety-valve, constructed like those connected with ordinary steam engines, by a weight and gauge, self-regulated to any degree of density and pressure which may be thought best. The amount of fresh air which will pass through the bath will vary from fifty to one hundred cubic feet per minute, of any required density, varying from one and a half to three atmospheres or more; requiring half an hour to raise the pressure to the point required, and the same period to remove it; the patient remaining in the air-chamber two hours for the "*tonic bath*" of 70° F., and a shorter period for the "*sweating bath*" of 100° and upwards.

The establishment at Rochester, it may be remarked, is constructed after a similar fashion, and as the patient comes out of the bath one or two pails of tepid or cold water are dashed over him, and he is then briskly rubbed all over with a rough bath-towel, which leaves the skin in a soft, pliable condition, and with agreeable sensations.

*Physiological Effects.*—For such knowledge as I have of these, I am indebted to several intelligent persons who have undergone the treatment both at Toronto and Rochester; and of course such knowledge must be, to a very considerable degree, imperfect, lacking that accuracy and definiteness of statement so necessary in scientific matters. All, however, who have made careful observations, testify that the pulse becomes slower and fuller, and the respiration also slower; while there is a general feeling of strength, comfort and well-being, while they remained in a bath of from 20 to 25 pounds to the square inch, or that of one and a half to two atmospheres; provided the temperature was not much above that of 70°. F. The effects, however, vary somewhat in different individuals. When the temperature is raised to 90° and above, (and it should have been mentioned, that by means of coils of steam-pipe the temperature of the rooms can be raised to any required point, though I have met with no one who has entered the bath heated above 114° F.) it is agreed on all hands, that it proves powerfully sudorific; the water pouring in streams from all parts of the body, and from the head and face so copiously as to require the constant use of a towel, which in a few minutes becomes so saturated as to require to be wrung out. The secretions, generally, particularly those of the skin, liver and kidneys, are increased, in a marked degree, both by the tonic and the sweating-bath; although by the latter, those of the skin in a vastly increased proportionate degree. The blood is powerfully drawn by it to the peripheral parts of the body, the veins become full and swollen; a ringing, painful sensation is experienced in the ears, which gradually becomes lessened as the pressure is increased, and usually returning again while the pressure is being removed, although the urine is generally copious, it is higher colored than natural, while the appetite and digestion usually undergo a marked improvement. The effects of the "tonic" and "sweating-bath" vary, however, very much, and also in different individuals. Some represent the muscular strength to have increased from the first, whether either bath was employed; while others state that the bath of 90° was followed by a sense of weakness and exhaustion, which it took a considerable time to recover from. Some, however, who have employed the Turkish bath (air at a high temperature, with shampooing,) state that they experienced none of that languor and muscular weakness from the compressed hot-air bath, which usually attends the operation of the former, or even that of the warm vapor, or hot-water bath. This has been attributed to the continued current of pure fresh air, containing double the quantity, or more, of oxygen, passing every moment through the air-chamber. The statements of Prof. Parke's, in his excellent work on "Military Hygiene," (1865,) in regard to the effects of compressed air-baths, vary somewhat from the above; inasmuch, as he says, that evaporation from the lungs and the surface, is less than ordinary; a result entirely different from what we should, reasoniag, *a priori*, expect.

*Curative Effects.*—Of these, also, I have but a very limited amount of knowledge, knowing the physiological effects, we might be expected, perhaps, to predicate to some extent, at least, what its cnrative influence must be in particular forms of disease; but we know too little in regard to the former to justify us in arousing any very satisfactory or well-founded conclusins in regard to the latter. That it is a powerful agent for good or evil, according to the judgment and skill with which it is managed, there can be no question. There is too much reason to fear, however, that as it has thus far, for the most part, in this country been in the hands of the uneducated and empirics, unacquainted both with physiology and pathology, it has been the means of accomplishing more evil than good. Indeed, from the statements of those who have had the best opportunities of observation, and have daily witnessed or experienced its effects, for weeks together, it has been so varied in its action, under apparently similar conditions, that it is very difficult to classify the results, even in the most general manner.

According to a private circular, issued from the Rochester Institution, this treatment has been very successful in both acute and chronic rheumatism; in different forms of dyspepsia; in congestions and torpid conditions of the liver and spleen; in nervous affections generally, particularly in neuralgias and hypochondriasis and nervous prostration, with symptoms of threatened insanity; in amenorrhœa and retention of the menses: in asthma and chronic bronchitis; and in constipation and incipient diseases of the lungs, etc. Some of the cases of cures reported, especially of chronic rheumatism, attended with contraction of the limbs and deformity, are so remarkable, as to border almost on the miraculous. Reasoning from its known physiological effects, we should be inclined to think it might be used to advantage in relieving internal inflammations and congestions, such as engorgements of the liver and spleen; also spasmodic asthma, and other affections, characterized by undue determinations of blood to particular organs. To what degree the effects would be *permanent*, remains for future observations to settle; and on this much of its real therapeutic value must depend. It is evident that under a pressure of two atmospheres the blood must, in a given period of time, be brought in contact with a double amount of oxygen, and that this must exert an important influence upon the functions of sanguinification, nutrition, calorification, secretion, etc. An equal proportion, too, of carbonic acid gas would escape from the lungs, and thus every organ and tissue of the body experience a more profoundly stimulating and vitalizing influence. It can scarcely be doubted, moreover, that under such an amount of pressure the capillary circulation generally would be more active and perfect, and that such a bath would accomplish, in a good degree, what is the ordinary effect of exercise, without the existence or fatigue attending it.

It is now nearly forty years since M. Junall, an able Parisian practitioner, devised an apparatus by which, as he assures us, the atmospheric pressure on the body may be increased from 26,000 pounds, which he states to be its ordinary amount, to one hundred thousand pounds. This he calls a *pneumatic bath.* We have not the means at hand which would enable us to describe his apparatus; but he employed it, he says, with advantage in diseases of the chest and larynx, in anorexia, scrofula, marasmus, certain forms of deafness, and diseases of the skin, arising from debility of this organ.

The profession has long been in the habit of employing M. *Junad's boat* in cases of shrinking, or dwindling of the extremities, for the purpose of increasing the nutrition of the affected limb, and with the best results. While *atmospheric pressure* seems to have its advocates in this region, in the treatment of diseases; the opposite, or "*pneumatic occlusion*," as it is called, is coming in fashion among the Parisians. The distinguished French Surgeon, M. Guorin, lately read a paper before the "Academy of Siciences" in Paris, upon the use of an apparatus for *keeping the skin free from the contact of air.* His plan includes, 1st, an exhausting air pump; 2d, vulcanized caoutchous "mufflers" (*manchous*) of suitable form for the parts to be acted upon. The opening of the envelope has a diameter about two centimetres less than the diameter of the surface it is to surround, so that by means of moderate elastic pressure all communication with the external air can be cut off. Each *muffler* is furnished with one or more india-rubber tubes connecting the confined spaces with the receiver of the air pump. 3d, a number of envelopes made of some permeable material of varying thickness, which are to be placed between the impermeable muffler and the tegumentary surface, and are intended to facilitate the removal of vaporite, by air pump. If the hand, for [example, is to be acted upon, it is first enveloped in cotton-wool, then placed in the india-rubber muffler, which is closed by an elastic band around the wrist. The tube is then connected with exhaustive receiver, so that when the tap is turned a vacuum is formed, and the enveloping membrane "glues" itself over the surface, and maps out like a second skin, the form at the fingers, and the marks upon their surfaces.

The idea of M. Guorin has evidently been borrowed from M. Junad, who invented the *pneunatic boot*, to which reference has been made, and which is so highly recommended by Brown-Séquard, and others. That M. Guerin's apparatus would be very effectual causing the blood to be forced into the minute capillaries of the part subjected to the treatment, thus favoring nutrition, there can be no doubt whatever, and we think it may therefore prove efficacious especially in cases of muscular atrophy in any part of the body. It seems rather remarkable, however, that exactly opposite processes of treatment, viz: *increased and diminished pressure*, should accomplish the same therapeutical effects. But when we consider that both tend to draw the blood powerfully to the peripheral surfaces, and thus relieve internal congestions, the mode of cure is sufficiently evident. M. Guorin's *apparatus* would seem to be only a more general and efficient method of carrying out the principle of *dry-cupping*. This apparatus has been employed by Dr. Brown-Séquard, in the "Royal Paralytic and Epileptic Hospital" of London, as well as in his private practice, with the most marked success in similar cases, and Dr. Dewey of New York, states, (*New York Journal of Medicine*) that he has frequently employed it in paralysis, especially hemiplegia, in connection with electricity, with the most decided benefit. In this apparatus, as is generally well known, the air is exhausted around the affected limb by a small pump attached to the upper portion of the boot, and thus the blood is forcibly driven into the limb by atmospheric pressure, resulting in its rapld nutrition and consequent increase of size. Brown-Sèquard states that the result is a marked increase of muscular irritability, as well as increase of the nutritive processes. Reasoning from analogy, is it not a legitimate conclusion that what takes place in a portion of the body from atmospheric pressure, may also occur in the whole body, under similar conditions?

These suggestions and remarks, gentlemen, are offered rather for the purpose of stimulating enquiry, than from any positive value, which I am disposed to attach to them. The subject I believe is worthy of investigation by the scientific members of our profession. The practice by means of general compressed air-baths has been too long in the hands of empirics and irregular practitioners. Scientific and legitimate medicine professes to appropriate all positive means of cure-all remedial agents calculated to relieve human suffering or cure disease. Let us not disdain to gather knowledge, no matter from what source it may come. It little becomes us, as members of a learned and liberal profession, to refuse to enquire into the merits of any particular method of treatment because it has been employed by illiterate men. This particular rêmedy of compressed air, however, as we have seen, was first introduced to the knowledge of the world by men distinguished in science, and known for their zeal and medical knowledge. In this country, at least, it has been allowed to fall into the hands of laymen and empirics. It is time an effort should be made to rescue it from such hands; to study its real value; to investigate more completely its effects; and thus place it among the recognized resources of our art.

